# Alterations of Calcium Channels in a Mouse Model of Huntington’s Disease and Neuroprotection by Blockage of Ca_V_1 Channels

**DOI:** 10.1177/1759091419856811

**Published:** 2019-06-19

**Authors:** Artur S. Miranda, Pablo Leal Cardozo, Flavia R. Silva, Jessica M. de Souza, Isabella G. Olmo, Jader S. Cruz, Marcus Vinícius Gomez, Fabiola M. Ribeiro, Luciene B. Vieira

**Affiliations:** 1 Department of Biochemistry and Immunology, ICB, Universidade Federal de Minas Gerais, Belo Horizonte, Brazil; 2 Department of Neurotransmitters, IEP, Santa Casa, Belo Horizonte, Brazil; 3 Department of Pharmacology, ICB, Universidade Federal de Minas Gerais, Belo Horizonte, Brazil

**Keywords:** BACHD mice, calcium channel blockers, Cav1 channels, Huntington’s disease, neurodegeneration, neuroprotection

## Abstract

Huntington’s disease (HD) is a neurodegenerative autosomal dominant disorder, characterized by symptoms of involuntary movement of the body, loss of cognitive function, psychiatric disorder, leading inevitably to death. It has been previously described that higher levels of brain expression of Ca_v_1 channels are involved in major neurodegenerative disorders, such as Alzheimer’s disease and Parkinson’s disease. Our results demonstrate that a bacterial artificial chromosome (BAC)-mediated transgenic mouse model (BACHD mice) at the age of 3 and 12 months exhibits significantly increased Ca_v_1.2 protein levels in the cortex, as compared with wild-type littermates. Importantly, electrophysiological analyses confirm a significant increase in L-type Ca^2+^ currents and total Ca^2+^ current density in cortical neurons from BACHD mice. By using an *in vitro* assay to measure neuronal cell death, we were able to observe neuronal protection against glutamate toxicity after treatment with Ca_v_1 blockers, in wild-type and, more importantly, in BACHD neurons. According to our data, Ca_v_1 blockers may offer an interesting strategy for the treatment of HD. Altogether, our results show that mutant huntingtin (mHtt) expression may cause a dysregulation of Ca_v_1.2 channels and we hypothesize that this contributes to neurodegeneration during HD.

## Introduction

Huntington’s disease (HD) is a progressive neurodegenerative disorder characterized by symptoms that include involuntary movement of the body, loss of cognitive function, psychiatric disorders, and inevitable death (Ehrnhoefer et al., 2009, Labbadia and Morimoto, 2013). HD is an autosomal dominant disease caused by poly-glutamine expansion in a protein named huntingtin (Htt), leading to aggregate formation, as in a typical case of protein misfolding (Li and Li, 2004). However, the molecular mechanisms linking Htt mutation and neuronal cell death have not yet been fully elucidated (Davies et al., 1997, Tobin and Signer, 2000, Labbadia and Morimoto, 2013). The development of HD is determined by the progressive neuronal cell death occurring in the neocortex and striatum of affected patients (DiFiglia, 1990). There are some data showing that the neuronal cell loss that takes place in HD is due to glutamatergic excitotoxicity, which is mediated by excessive influx of intracellular Ca^2+^ (DiFiglia, 1990, Calabresi et al., 1999). Several studies indicate that mHtt promotes Ca^2+^ signaling alterations, which might be closely associated with the death of striatal neurons (Oliveira et al., 2006, Rosenstock et al., 2010, Wu et al., 2016, Raymond, 2017). It has been described that high levels of intracellular Ca^2+^ produce abnormalities in the central nervous system that might be associated with voltage-gated Ca^2+^ channels (Sattler and Tymianski, 2000, Cano-Abad et al., 2001, Luo et al., 2005, Swayne et al., 2005, Kim et al., 2008, Min et al., 2013). Alterations of voltage-gated channels may play an important part in disorders such as Alzheimer’s disease (AD), Parkinson’s disease (PD), epilepsy, and ischemia (Kim and Rhim, 2004, Pierrot et al., 2004, Luo et al., 2005, Swayne et al., 2005, Wu et al., 2011). Furthermore, dysfunction of L-type Ca^2+^ channels has been implicated in some of the most prevalent neurodegenerative diseases, including AD and PD (Hurley et al., 2013, Min et al., 2013). Notably, previous studies have demonstrated that high levels of Ca_v_1.2 channel expression in the brain are involved in AD (Kim and Rhim, 2011), and additional data showed that isradipine, an L-type Ca^2+^ channel blocker, offers neuroprotection in a mouse model of PD (Ilijic et al., 2011). Therefore, we attempted to investigate whether Ca_v_1 channels played a role on HD using a bacterial artificial chromosome (BAC)-mediated transgenic mouse model, BACHD (Gray et al., 2008). BACHD mice express full-length human Htt, exhibit progressive motor deficits, and late-onset selective neurodegeneration in the cortex and striatum. Accordingly, this mouse model is well suited for therapeutic trials (Ehrnhoefer et al., 2009).

Our results show that Ca_v_1.2 protein levels are increased in the cortex of BACHD mice at 3 and 12 months of age. On the other hand, BACHD mice at 12 months did not show any alterations of Ca_v_1.3 protein levels in the cortex, hippocampus, and striatum tissue. Interestingly, Ca_v_1.2 mRNA levels were not different when comparing WT and BACHD mice, indicating that changes in protein levels are not due to gene expression alterations. Furthermore, whole-cell electrophysiology recordings from cortical BACHD neurons show an increase in L-type Ca^2+^ currents and also in Ca^2+^ current density, as compared with WT. In addition, we performed an assay using nifedipine and isradipine, which are L-type Ca^2+^ channel blockers, to assess the neuroprotective effect of these drugs following glutamate insult. Our data showed that after applying the tested Ca_v_1.2 channel blockers, there was a decrease in neuronal cell death in both WT and BACHD cultured neurons, even though cell death levels were higher in BACHD neurons. Notably, our results indicate the set point theory that calcium homeostatic mechanisms, including Ca_v_1.2 channels-mediated influx, regulate the intracellular Ca^2+^ levels at or near an optimal set point. Thus, different concentrations of Ca_v_1.2 channel blockers appeared to stabilize the free cytosolic Ca^2+^ concentration at optimal levels, even if they incompletely suppressed Ca^2+^ influx. Altogether, these data suggest a previously unrecognized mechanism, where an increase in protein levels and function of Ca_v_1.2 channels may lead to enhanced levels of intracellular Ca^2+^, which ultimately could damage irreversibly the neurons and contribute to HD pathogenesis.

## Material and Methods

### Ethics Statement

All procedures used in this study were approved and strictly followed the ethical principles of animal experimentation adopted by the Ethic Committee on Animal Use of Federal University of Minas Gerais and institutionally approved under protocol number 139/2013.

### Animals

Mice were housed in an animal care facility at 23°C on a 12-h light/12-h dark cycle with food and water provided ad libitum. C57/BL6 mice (25–30 g) were purchased from the animal facility (CEBIO) located at the Universidade Federal de Minas Gerais. FVB/NJ wild-type (WT) and FVB/N-Tg (Htt*97Q) IXwy/J (BACHD) (Gray et al., 2008) were purchased from The Jackson Laboratory (Bar Harbor, ME, USA). BACHD mice is a transgenic mouse model expressing full***-***length human Htt, exhibiting progressive motor deficits and late-onset selective neurodegeneration in the cortex and striatum.

## Reagents and Chemicals

Neurobasal medium, N2 and B27 supplements, and GlutaMAX (50 µg/ml penicillin and 50 µg/ml streptomycin) were purchased from Thermo Fisher Scientific. ECL Luminol Prime, G-Sepharose and Neutravidin beads were purchased from GE Healthcare. Anti-β-actin primary antibody (RRID:AB_476697), nifedipine, isradipine, protease inhibitors and all the other reagents were purchased from Sigma-Aldrich. Mouse Anti-Ca_v_1.2 calcium channel Monoclonal Antibody, was purchased from Millipore (RRID:AB_10807024); Mouse Anti-Ca_V_1.3 (CACNA1D) Antibody was purchased from Alomone (RRID:AB_2039775).

### Neuronal Primary Culture Preparation

Neuronal cultures were prepared from the cortical region of either WT or BACHD mouse embryo brains, both genders, at the embryonic Day 15 (E15), as described previously (Doria et al., 2013). After dissection, cortical tissue was submitted to trypsin digestion followed by cell dissociation using a fire-polished Pasteur pipette. Cells were plated on poly-L-ornithine coated dishes in Neurobasal medium supplemented with N2 and B27 supplements, 2 mM GlutaMAX, 50 µg/ml penicillin, and 50 µg/ml streptomycin. Cells were incubated at 37°C and 5% CO_2_ in a humidified incubator and cultured for 8 to 12 days *in vitro* with medium replenishment every 4 days. Previous data showed that our cultures are almost pure neuronal cultures, as 99.5% of the cells are neurons, 0.5% are microglia, and 0% are astrocytes.

### Cell Death Assay

Neurons were incubated for 20 h in the presence or absence of L-type Ca^2+^ channel blockers or 50 μM glutamate, as indicated in the Table 1. Cell death was determined by live or dead viability assay, as described previously (Doria et al., 2013). Briefly, neurons were stained with 2 mM calcein acetoxymethyl ester (AM) and 2 mM ethidium homodimer-1 for 15 min and the fractions of live (calcein AM positive) and dead (ethidium homodimer-1 positive) cells were determined. Neurons were visualized by fluorescence microscopy using a Floid Microscope (Life Technologies). Cells were analyzed per well in triplicate using ImageJ™ software. Dead cells were expressed as a percentage of the total number of cells.

**Table 1.** Summary of Cell Death Assay.

Corticostriatal, cultures 8–12 DIV	WT	BACHD
Pretreatment	Nifedipine (0, 0.1, 1, 10 nM )	Nifedipine (0, 0.1, 1, 10 nM)
Pretreatment	Isradipine (0, 0.1, 1, 10 nM)	Isradipine (0, 0.1, 1, 10 nM )
Glutamate insult (50 mM)	+	+

*Note.* WT = wild type; DIV = days *in vitro*; BACHD = bacterial artificial chromosome (BAC)-mediated transgenic mouse model.

## Immunoblotting

The cortex, hippocampus, and striatum of BACHD and WT mice were dissected and lysed in RIPA buffer containing protease inhibitors. A total of 100 µg of total cellular protein for each sample was subjected to sodium dodecyl sulfate-polyacrylamide gel electrophoresis, followed by electroblotting onto nitrocellulose membranes. Membranes were blocked with 10% skim milk in wash buffer (150 mM NaCl, 10 mM Tris-HCl, and 0.075% Triton X 100, pH 7.4) for 1 h and then incubated with rabbit anti-Ca_v_1.2 (1:200), anti-Ca_v_1.3 (1:200), or mouse anti-β-actin (1:1.000) antibodies in wash buffer containing 3% skim milk overnight at 4°C. Membranes were rinsed 3 times for 5 minutes with wash buffer and then incubated with either secondary horseradish peroxidase-conjugated goat anti-rabbit IgG (1:5.000) or secondary horseradish peroxidase-conjugated goat anti-mouse IgG (1:5.000) in wash buffer containing 3% skim milk for 1 h at room temperature. Membranes were rinsed 3 times for 10 minutes with wash buffer and incubated with ECL luminol Prime. Nonsaturated, immunoreactive bands were quantified by scanning densitometry using Image Quant LAS software (GE Healthcare). Immunoband intensity was obtained by ImageJ™ software. Ca_v_1.2 levels were normalized to actin levels.

### RT-qPCR

Total RNA from 2- and 12-month-old WT and BACHD (Q97) mice was isolated using TRIzol™ reagent, according to manufacturer’s instructions (Thermo Scientific). Then, RNA was resuspended in 12 µL of Nuclease-free water and quantified by absorbance at 260 nm in a spectophotometer (Multiskan GO, Thermo Scientific). Two micrograms of total RNA were reverse-transcribed in a 20 µL reaction volume, the generated cDNA diluted 10× and quantitative PCR performed using Power SYBR™ Green PCR Mix (Applied Biosystems) in the QuantStudio™ 7 Flex Real-Time PCR Platform (Applied Biosystems). RT-qPCR was carried out to detect mRNA of the following genes: L-type Calcium channels, L-type, alpha 1C subunit (CACNA1C, which encodes Ca_V_1.2; forward: 5′-CATCACCAACTTCGACAACTTC-3′; reverse: 5′-CAGGTAGCCTTTGAGATCTTCTTC-3′); and alpha 1D subunit (CACNA1D, which encodes for Ca_V_1.3; forward: 5′-GCTCGGTGGCTGTATTTTCA-3′; reverse: 5′-ATCGGGCATCAGTCTCTTGG-3′); 60S ribosomal protein L32 (RPL32; forward: 5′- GCTGCCATCTGTTTTACGG-3′; reverse: 5′-TGACTGGTGCCTGATGAACT-3′); and actin (ACTIN, forward: 5’- TGGAATCCTGTGGCATCCATGA-3′; reverse: 5′-AATGCCTGGGTACATGGTGGTA-3′). All primers used in this study were validated by serial dilution, and reaction efficiency calculated and determined to range from 90% to 110% (data not shown). RT-qPCR data were calculated by 2-ΔCt method and normalized by the average of RPL32 and actin.

## Electrophysiology

Whole-cell voltage clamp recordings were obtained using an EPC-10 patch-clamp amplifier (HEKA, Holliston, Massachusetts) at room temperature (22°C–25°C). Current recordings were filtered at 2.9 kHz and digitally sampled at 10 kHz. Patch pipette resistance was 2.5 to 3.0 MΩ and was filled with an internal solution composed of (120 mM CsCl, 1 mM MgCl_2_.6H_2_O, 10 mM HEPES, 10 mM EGTA, 2 mM Mg-ATP, pH 7.2). Cs^+^ was used in the pipette solution to block voltage-dependent K^+^ channels, as well as to increase membrane resistance (typically above 100MΩ), improving voltage-clamp quality. Ba^2+^ was used as charge carrier in all experiments. Primary cultured cortical neurons with series resistance over 8.0 MΩ were excluded from the analysis. During whole-cell experiments, neurons were bathed in Tyrode solution (140 mM NaCl, 5.4 mM KCl, 0.5 mM MgCl_2_, 0.33 mM NaH_2_PO_4_, 1.8 mM CaCl_2_, 5 mM HEPES, 11 mM glucose, pH 7.4). After the establishment of the whole-cell configuration, the plates containing cells were perfused with control barium solution (130 mM TEA-Cl, 2 mM MgCl_2_.6H_2_O, 10 mM BaCl_2_, 10 mM HEPES, 10 mM Glucose, pH 7.2) for 5 min followed by 5 min exposure to nifedipine 5 μM added to the same solution. Cortical neurons were hyperpolarized to −100 mV for 50 ms from a holding potential of −80 mV, followed by a ramp protocol from −100 mV to +50 mV at a rate of 1.5 V/s, with a frequency of 0.1 Hz. L-type Ca^2+^ currents were determined by digital subtraction between Ca^2+^ currents before and after the effects of nifedipine. We observed a significant rundown in four WT and three BACHD recorded neurons. Extent of rundown magnitude varied between 30% and 80% of total peak initial current, measured 300s after beginning of stimulation protocol. Also, the electrophysiological experiments were always age matched.

## Statistical Analyses

**Figure 1.**
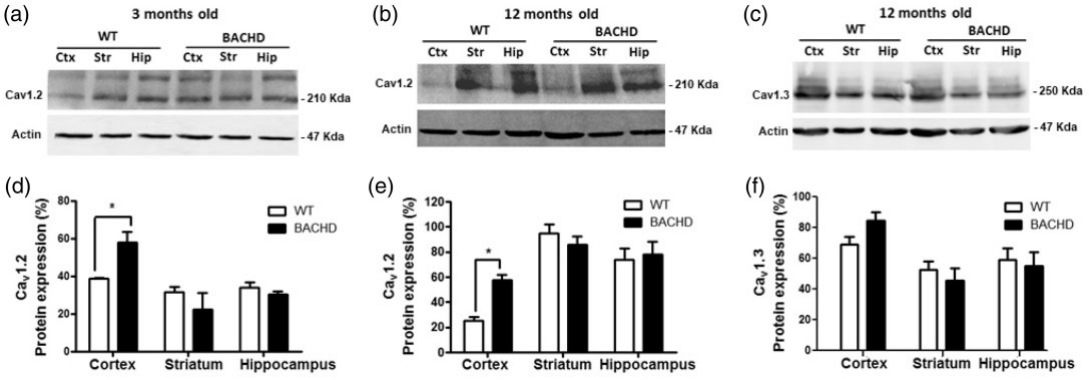
Ca_v_1.2 levels are increased in the cortex of 3- and 12-month-old BACHD mice. Shown are representative immunoblots for Ca_v_1.2 or Ca_v_1.3 (upper panel) and actin (lower panel) expression in the cortex, striatum, and hippocampus of either WT or BACHD mice at 3 (a) and 12 (b and c) months of age. Graphs show the densitometric analysis of total levels of Ca_v_1.2 or Ca_v_1.3 in the cortex, striatum, and hippocampus of either WT or BACHD mice at 3 (d) and 12 (e and f) months of age. A total of 100 μg of protein from cell lysates were was used for each sample. Data represent the means ± SEM of four independent experiments, expressed as percentage of actin levels. *indicates significant difference as compared with WT Ca_v_1.2 levels (*p* < .05).

**Figure 2.**
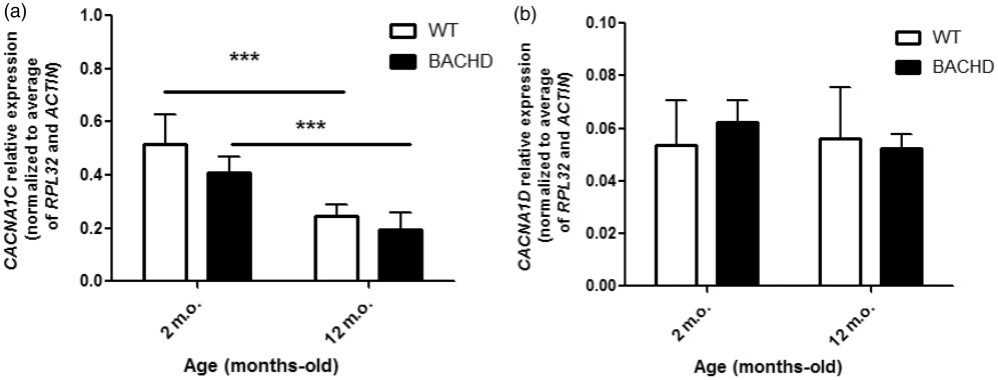
mRNA of Ca_v_1.2 and Ca_v_1.3 are not altered in the cortex of 2- and 12-month-old BACHD mice. Graphs show mRNA of Ca_v_1.2 (A) and mRNA of Ca_v_1.3 (B), in the cortex, of either WT or BACHD mice at 2 and 12 months of age. mRNA levels were assessed by quantitative RT-PCR, which was performed in triplicate and normalized to RPL32 mRNA levels and actin. Data represent the means ± SEM, *n*  = 6. * indicates significant differences (*p*  < .05).

**Figure 3.**
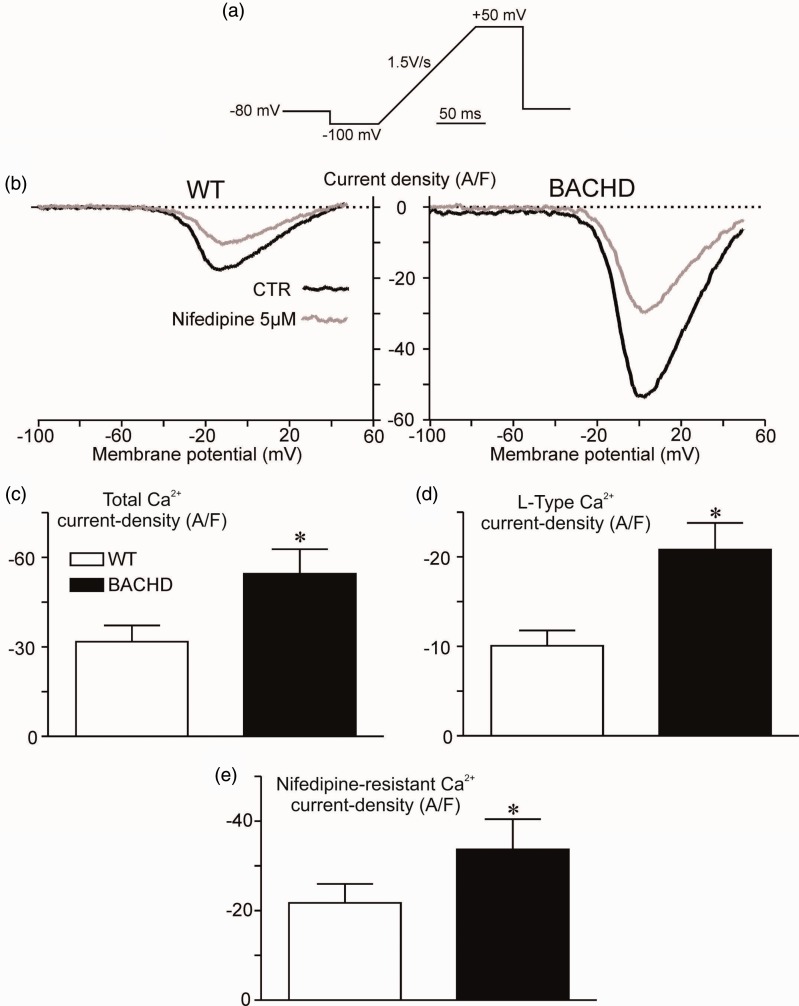
L-type Ca^2+^ currents are increased in BACHD cortical neurons. (a) Voltage-clamp ramp protocol used to record Ica currents. (b) Representative records of the Ca^2+^ current–voltage relationship from WT (left panel) and BACHD (right panel) in the absence (black) or after exposure to nifedipine 5 μM (gray). (c) Graphs show peak total Ca^2+^ current-density and (d) L-type Ca^2+^ current-density from WT and BACHD mice. (e) Graphs show peak total Ca^2+^ current-density after exposure to nifedipine 5 μM from WT (*n* = 8) and BACHD (*n* = 7) mice. *indicate significant differences as compared with matched WT (*p* < .05).

**Figure 4.**
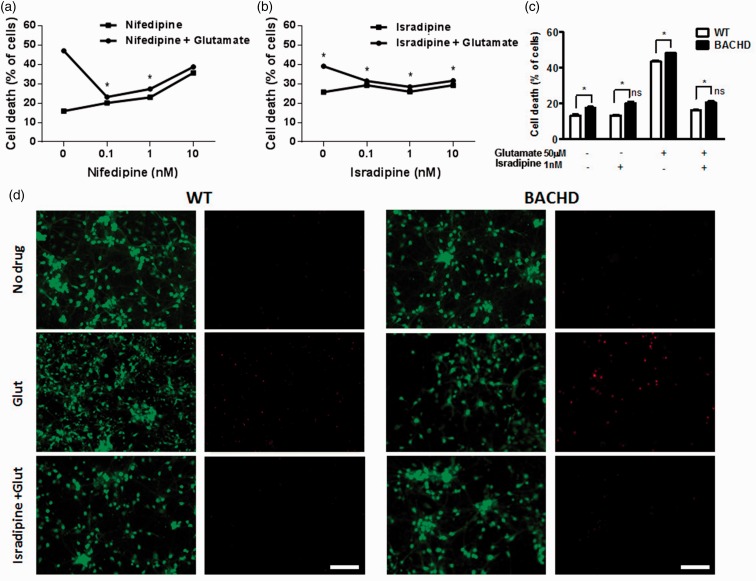
Selective L-type Ca^2+^ blockers protect against glutamate-induced neuronal cell death. (a and b) Graph shows percentage of neuronal cell death of primary cultured corticostriatal of C57/BL6 mice neurons that were either untreated or treated with 0.1 nM, 1 nM or 10 nM of (a) nifedipine or isradipine (b) in the presence or absence of 50 μM glutamate for 20 h (closed circles). (c) Graph shows percentage of neuronal cell death of primary cultured corticostriatal of WT (FVB/NJ) or BACHD mice neurons that were either untreated or treated with 1 nM of isradipine in the presence or absence of 50 μM glutamate for 20 h. (d) Shown are representative images for primary cultured corticostriatal neurons obtained from either WT (FVB) or BACHD embryos that were either untreated or treated with 50 μM glutamate or 50 μM glutamate plus 1 nM isradipine for 20 h and labeled with calcein AM (green, live cells) and ethidium homodimer-1 (red, dead cells). Scale bar = 100 μM. Data represent the means ± SEM of four independent experiments. * indicates significant difference as compared with WT for each condition and *ns* indicates no significant difference as compared with untreated neurons (basal cell death) (*p* < .05).

Data were tested for normality using Shapiro–Wilk Test and the results indicated normal distribution of data. Means ± standard error of the mean (SEM) are shown for the number of independent experiments indicated in Figure 1, 2, 3 and 4. GraphPad Prism™ software was used to analyze data for statistical significance determined by either unpaired *t* test (for comparing two groups) or two-way analysis of variance testing followed by Bonferroni post hoc multiple comparison testing.

## Results

### BACHD Mice Exhibit Increased Cortical Levels of Ca_v_1.2 Protein

It has been demonstrated that Ca_v_1 protein expression is increased in some neurodegenerative disorders, including AD and PD (Ueda et al., 1997, Hurley et al., 2013, 2015). Thus, we investigated whether Ca_v_1.2 or Ca_v_1.3 protein levels are altered in BACHD mice. For that, we performed western blotting experiments to measure Ca_v_1.2 or Ca_v_1.3 levels in total cell lysates (Figure 1(a) to (c)). Our results showed a significant increase in Ca**_v_**1.2 levels in the cortex, but not in the hippocampus or striatum, of 3-month-old BACHD, as compared with WT littermates (*t*_4_=3.393 *p* = .0146; Figure 1(d)). Moreover, Ca_v_1.2 levels in 12-month-old BACHD mice were significantly increased in the cortex, but not in the hippocampus or striatum, as compared with WT mice (*t*_4_=6.297, *p* = .0007; Figure 1(e)). Importantly, our immunoblotting analyses showed that Ca_v_1.3 protein levels were not altered in the cortex, hippocampus, or striatum of 12-month-old BACHD mice, as compared with WT littermates (*t*_4_ = 4.175, *p* = .4103; Figure 1(f)). This increase in Ca_v_1.2 protein levels in the cortex could be due to either increased gene expression or decreased protein degradation. To test whether Ca_v_1.2 expression was increased in BACHD mice, we performed RT-qPCR to measure Ca_v_1.2 mRNA levels. Interestingly, Ca_v_1.2 (Figure 2(a)), as well as Ca_v_1.3 (Figure 2(b)) mRNA levels were not different when comparing WT and BACHD mice at 2 and 12 months of age. Therefore, it is possible that other posttranslational alterations, such as cell trafficking and protein degradation, could be responsible for Ca_v_1.2 increased protein levels.

### BACHD Neurons Display Increased L-Type Ca^2+^ Currents

Whole-cell patch-clamp experiments were conducted in primary cultured cortical neurons to evaluate whether enhanced Ca_v_1.2 protein levels would reflect in an increase in L-type Ca^2+^ current density. By digital subtraction of the total Ca^2+^ current before and after exposure to 5 μM of nifedipine, we assessed L-type Ca^2+^ current (nifedipine-sensitive current). We used 5 μM nifedipine to access the full effect on Ca_v_1.2 channels. Figure 3(a) displays the ramp protocol used in patch-clamp experiments. Representative records of the current–voltage relationship of WT (left panel) and BACHD (right panel) cortical neurons before and after the exposure to 5 μM nifedipine are displayed in Figure 3(b). It is evident that both total Ca^2+^ peak current density (−31.74 ± 5.4 pA/pF vs. −54.44 ± 8.3 pA/pF; WT vs. BACHD; Figure 3(c)) and L-type Ca^2+^ peak current density (−10.05 ± 1.7 pA/pF vs. −20.8 ± 2.9 pA/pF; WT vs. BACHD; Figure 3(d)) were increased in BACHD cortical neurons, as compared with WT cortical neurons. Nifedipine was able to decrease Ca^2+^ peak current from both WT and BACHD neurons; however, nifedipine-resistant Ca^2+^ current was still higher in WT compared with BACHD, suggesting that a different voltage-gated Ca^2+^ channel may contribute to the putative increase in Ca^2+^ current density observed in cortical neurons from BACHD mice (Figure 3(e)). Cell capacitance was averaged at 36.9 ± 3.6 pF, *n* = 8 for WT and 37.3 ± 4.1 pF *n* = 7 for BACHD and did not differ from each other. Membrane resistance was estimated for each cell and multiplied by its respective capacitance to obtain τ values estimation (2.34 ± 0.44 ms for WT vs. 2.99 ± 0.62 ms for BACHD), which did not differ from each other. Altogether, these data strongly indicate that there is more Ca_v_1.2 activity in BACHD cortical neurons, which per se could underlie increased intracellular Ca^2+^ levels and, consequently, cell death. Indeed, our group have previously assessed the nifedipine-resistant Ca^2+^ current presented in Figure 3(e). Importantly, we observed a twofold increase in Ca_v_2.2 currents from BACHD-derived neurons that match the fraction of residual increase in total Ca^2+^ current apart from the Ca_v_1.2-dependent increase demonstrated in this article (Silva et al., 2017).

### Ca_v_1.2 Channel Blockers Protect Against Glutamate-Induced Neuronal Cell Death in BACHD Cultured Neurons

To test whether the antagonism of Ca_v_1.2 channels could be efficient to promote neuroprotection, we performed an *in vitro* assay to measure neuronal cell death. Primary cultured corticostriatal neurons from C57/BL6 (Figure 4(a) and (b)), FVB/NJ (WT; Figure 4(c)) or BACHD mouse embryos exposed to 50 μM glutamate for 20 h exhibited high level of neuronal cell death (Figure 4(c)). To establish a concentration–response curve, C57/BL6 neurons were incubated with glutamate in the presence of increasing concentrations (0.1, 1, and 10 nM) of nifedipine (Figure 4(a)) or israpidine (Figure 4(b)), and, as observed, neuronal cell death was significantly decreased. Interestingly, nifedipine at 10 nM was not able to promote neuroprotection. In fact, 10 nM nifedipine triggered neuronal cell death even in the absence of glutamate (Figure 4(a)). In addition, the most common adverse effect associated with nifedipine is reflex tachycardia secondary to pronounced vasodilatation. Some patients may experience symptoms of hypotension and flushing, as well as some severe adverse affects, including retinal ischemia, cerebral vascular accident, and myocardial ischemia and infarction (Murakami et al., 1972). Nifedipine and isradipine are examples of calcium channel blockers with 1,4-dihydropyridine (DHP) scaffold. Even though effectively used in clinics for the treatment of hypertension, the binding mechanism to their target, the L-type Ca^2+^ channel, Ca_v_1.2, is not completely understood. As previously reported, dihydropyridines can bind L-type Ca^2+^ channels in an isoform-selective manner, which may explain some of the clinical differences (Sinnegger-Brauns et al., 2009). Thus, we settled to perform more experiments using isradipine that is also able to block Ca_v_1.2 channels and has been described as neuroprotective (Anekonda and Quinn, 2011, Ilijic et al., 2011). To test whether this Ca_v_1.2 channel blocker could also be efficient to protect neurons expressing mutant Htt, BACHD corticostriatal neurons were also tested and compared with WT neurons (Figure 4(c) and (d)). BACHD neurons exhibited increased basal neuronal cell death levels, as compared with WT neurons (Figure 4(c) and (d)). Likewise, BACHD neurons were also more susceptible to glutamate insult than WT neurons (Figure 4(c) and (d)). Notably, this excitotoxic effect of glutamate was blocked by 1 nM isradipine in both WT and BACHD neurons (Figure 4(c) and (d)). In addition, 1 nM isradipine diminished cell death induced by glutamate to the same levels as those of untreated neurons (Figure 4(c) and (d)). Overall, these data pinpoint that Ca_v_1.2 channel blockers are capable of preventing the death of WT and BACHD primary cultured neurons in response to glutamate insult.

## Discussion

### Calcium Dysfunction in Neurodegenerative Diseases

Ca^2+^ fluxes across the plasma membrane and between intracellular compartments play important roles in neuronal function, including cell survival, synaptic transmission, plasticity, and gene transcription (Berridge, 2004). In most neurodegenerative disorders, Ca^2+^ regulation processes are compromised leading the neuronal cells to endure synaptic dysfunction, impairment in plasticity, oxidative stress, apoptosis, and death (Hajnoczky et al., 2003, Orrenius et al., 2003). In HD, it is also observed that excitotoxicity mediated by excessive activation of glutamate receptors leading to an excessive increase in intracellular Ca^2+^ concentration (DiFiglia, 1990, Chen et al., 1999). Several studies indicate that mHtt promotes Ca^2+^ signaling alterations, which are closely associated with cell death of striatal neurons (Zeron et al., 2002, Ribeiro et al., 2010). For instance, it has been described that mHtt is able to sensitize NMDA receptors, increasing NMDA channel permeability to Ca^2+^ in striatal neurons (Chen et al., 1999, Sun et al., 2001). Also, mHtt protein promotes destabilization of mitochondria, decreasing the ability of this organelle to regulate Ca^2+^ levels (Panov et al., 2002, Choo et al., 2004). In addition, mHtt is able to sensitize the inositol trisphosphate receptor (InsP_3_R), increasing the release of Ca^2+^ from intracellular stores (Tang et al., 2003, Bezprozvanny, 2011). Moreover, it has been shown that signaling through ryanodine receptor, critical for skeletal muscle excitation–contraction coupling, is altered by mHtt in HD neurons (Chen et al., 2011). Indeed, authors showed a significant decrease in L-type Ca^2+^ channel conductance, leading to changes of Ca^2+^ turnover in skeletal muscle of R6/2 mice and suggesting that changes may be associated with muscle pathology in HD (Braubach et al., 2014). Therefore, mHtt-mediated increased intracellular Ca^2+^ levels play an important role in the neuronal cell death that takes place in HD.

Alterations in voltage-gated Ca^2+^ channels are also implicated in age-related neuronal dysfunctions (Ueda et al., 1997, Tai et al., 2011, Daschil et al., 2013, Hurley et al., 2015). Interestingly, a relationship between voltage-gated L-type Ca^2+^ channels and AD has already been demonstrated, as published data indicate that β-amyloid peptide is capable of interacting with voltage-gated L-type Ca^2+^ channels, altering channel activity and promoting an increase in the expression of this Ca^2+^ channel at the plasma membrane (Scragg et al., 2005, Kim and Rhim, 2011). Notably, L-type Ca^2+^ channel blockers were also found to have neuroprotective effects against Aβ-induced neuronal apoptosis in cultured rat cortical neurons (Yagami et al., 2004) and from amyloid precursor protein (APP)-induced neurotoxicity in neuroblastoma cells (Anekonda and Quinn, 2011). Despite these various studies relating voltage-gated L-type Ca^2+^ channel and neurodegenerative diseases, there is no evidence of whether these channels could play a role in the Ca^2+^ alteration that occurs in HD patients. Our results presented here suggest that Ca_v_1 channels are altered in a mouse model of HD.

### Alterations of Ca_v_1 Channels in a Mouse Model of HD

Our investigation started with the observation that Ca_v_1.2 total protein levels are increased in the cortex of 3- and 12-month-old BACHD mice. As Ca_v_1.2 proteins are mostly located postsynaptically at somatodendritic locations, these channels regulate neuronal excitability and are also known to be involved in translating synaptic activity into alterations in gene expression and neuronal cell death (Westenbroek et al., 1990; Murphy et al., 1991; Hell et al., 1993; Obermair et al., 2004; Helton et al., 2005; Wankerl et al., 2010). Our immunoblotting analyses and also RT-qPCR results showed that neither Ca_v_1.3 protein nor mRNA levels are altered. Ca_v_1.3 channels activate more rapidly and at more negative voltages than Ca_v_1.2, which allows them to contribute to the stabilization of upstate potentials and the control of neuronal firing (Koschak et al., 2001). Interestingly, whole-cell patch-clamp data indicated that this increase in Ca_v_1.2 protein levels in BACHD mice are accompanied by enhanced L-type Ca^2+^ current density. Altogether, these data may suggest that an increase in intracellular Ca^2+^ observed in HD could also be related to alterations in Ca_v_1.2 protein levels and activity. Thus, therapeutic agents aiming to diminish this increase in cytosolic Ca^2+^ levels, by partially blocking L-type Ca^2+^ currents, may play an important role in protecting corticostriatal neurons against Htt-mediated neuronal cell death. Dihydropyridines are commonly used to establish the contribution of L-type Ca^2+^ currents in several neuronal processes, but the efficiency of these blockers depends on membrane potential, channel state, and channel subtype (Hess et al., 1984; Holz et al., 1988). Notably, the results with nifedipine and isradipine are very intriguing, as both drugs are able to bind to either Ca_v_1.2 or Ca_v_1.3 binding pockets (Takahashi and Catterall, 1987; Sinnegger-Brauns et al., 2009). However, the contribution of Ca_v_1.2 and Ca_v_1.3 isoforms to L-type Ca^2+^ currents in different neurons is difficult to assess using pharmacological tools, as a consequence of partial selectivity of DHPs. Thus, to clarify the potential role of Ca_v_1.2 and Ca_v_1.3 channels in HD pathology by using electrophysiology protocols, newer selective blockers for those channels have to be developed. According to our results, the excitotoxic effect caused by high concentrations of glutamate in BACHD cultured neurons was diminished by adding 1 nM isradipine to the culture. Furthermore, 1 nM isradipine was able to reduce glutamate-induced neuronal cell death to the same levels as control. As observed in our whole-cell patch-clamp data, Ca_v_1.2 blockage did not completely abolish the increase in total Ca^2+^ current observed in BACHD neurons, suggesting that a different voltage-gated Ca^2+^ channels may play a part in HD dysfunction. As previously mentioned, our research group observed an increase in Ca_v_2.2 currents in BACHD derived neurons (Silva et al., 2017). Therefore, there are likely two components rendering the increase in total calcium current from BACHD cortical neurons compared with WT: a twofold increase in L-type Ca^2+^ currents observed in this study and a twofold increase in N-type Ca^2+^ currents (Silva et al., 2017). Interestingly, even though more subtypes of Ca^2+^channels seem to be affected by mutant Htt, the blockage of L-type Ca^2+^ currents by isradipine (0.1, 1, and 10 nM) was sufficient for completely rescuing glutamate-induced neuronal cell death, at least an *in vitro* setting using primary neuronal cultures. Nifedipine was also efficient to rescue neuronal cell death at the concentrations of 0.1 and 1 nM. However, 10 nM nifedipine did not prevent glutamate-induced neuronal cell death and was also neurotoxic even in the absence of glutamate. Thus, 0.1 to 1 nM of nifedipine appeared to stabilize the free cytosolic calcium concentration at optimal levels, even if it incompletely suppressed Ca^2+^ influx.

It is also important to emphasize that we used an *in vitro* assay to measure neuronal cell death against glutamate toxicity and neuroprotection after treatment with Ca_v_1 blockers. It is well known that cultures from embryonic neurons have substantially abnormal environment (Millet and Gillette, 2012), as in this case neuronal cultures are devoid of glial cells and, therefore, are not in the presence of many factors that could alter neuronal survival or cell death (Doria et al., 2013). Furthermore, although *in vitro* models are successfully used in biological fields to study biological and pharmacological mechanisms, extrapolation of the results to human are not possible. Thus, additional *in vivo* studies are needed in order to claim that blocking Ca_v_1channels could be neuroprotective.

**Figure 5.**
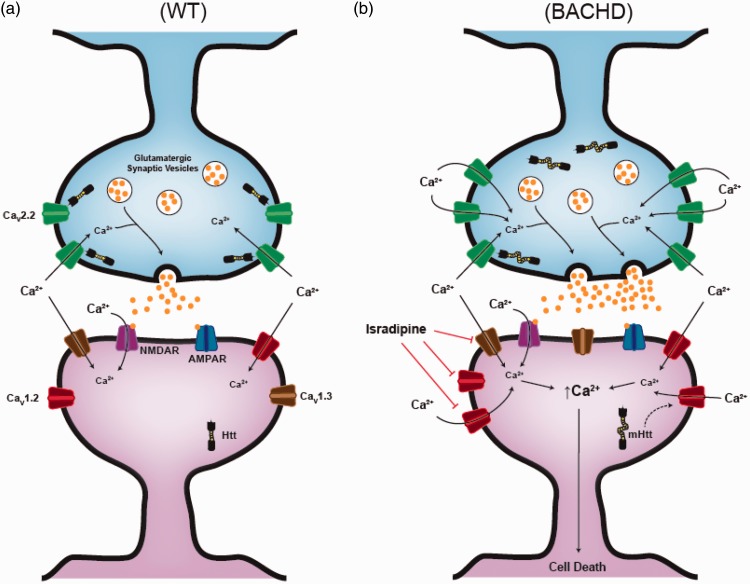
Hypothetical mechanism of Ca_v_ channels involved in neuronal death in a mouse model of HD. Cortical neuronal cultures obtained from BACHD mice (b) display elevated Ca_v_1.2 channel levels, but not Ca_v_1.3, compared with WT littermates (a). Taken together with previous observations (Silva et al. 2017), we hypothesize that (a) the disruption of mHtt interaction with Ca_v_2.2 channels increase plasma membrane levels, leading to a greater Ca^2+^ influx into the presynaptic compartment. This, in turn, (b) promotes a larger release of glutamate at the synaptic cleft, accentuating glutamate receptors activation, depolarizing the cell membrane, and opening Ca_v_1.2 channels in the postsynaptic compartment. In addition, (c) mHtt affects Ca_v_1.2 total protein levels either by altering protein trafficking or degradation; this enhancement of Ca_v_1.2 channel levels trigger elevated Ca^2+^ into the postsynaptic neuron and, lately neuronal death. Treatment with isradipine (b), a Ca_v_1 channel blocker, is able to reduce L-type Ca^2+^ currents *in vitro*, preventing the excitotoxic effects of glutamate insult and rescuing neuronal death in BACHD neurons to WT levels.

Another key point to be raised is whether glutamate induced calcium entry through Ca_v_1.2 channels may reflect what occurs in HD. We believe that Ca_v_1.2 channels may play a small piece in the puzzle of increasing intracellular Ca^2+^ observed in HD. Thus, we cannot discard the role of NMDA receptors, IP3 receptors, and others in inducing neuronal cell death (DiFiglia, 1990, Chen et al., 1999, Tang et al., 2003, Bezprozvanny, 2011). Maybe by blocking all other targets, we could drastically reduce neuronal cell death in BACHD neurons. However, blocking all Ca^2+^ influx may also interfere with the homeostasis of neuronal cells (Hajnoczky et al., 2003). Besides, literature has identified Ca_v_1.2 and Ca_v_1.3 channels as specific molecular and cellular cascades that underlie mood (anxiety and depression), social behavior, and cognition in rodents (Mogilnicka et al., 1987; Bader et al., 2011; Kabir et al., 2016, 2017) As well, interesting data demonstrated that the BACHD mouse recaps clinical HD with early psychiatric aspects, such as depressive and anxiety-like features (Hult Lundh et al., 2013). Likewise, more *in vivo* data are also required to demonstrate whether blocking Ca_v_1.2 is sufficient to delay or ameliorate the onset of behavioral deficits in BACHD mice. Consequently, we hypothesize that partially blocking Ca_v_1.2 channels could provide a better option for preventing neuronal cell loss, likely by modulating the overactivity of those channels (Figure 5).

### Potential Mechanisms Explaining mHtt Effects on Ca_v_1.2 Channels

Another remarkable question hoist by our data is how mHtt alters Ca_v_1.2 channel levels. It has been shown previously by our group and others that mHtt can interact with voltage-gated Ca^2+^ channels, such as Ca_v_2.2 (Swayne et al., 2005, Silva et al., 2017). Also, it seems that mHtt can regulate Ca_v_2.2 at many levels by altering channel levels, modifying Ca_v_2.2 interaction with its protein partners and changing channel activity (Silva et al., 2017). Interestingly, although Ca_v_1.2 protein levels are increased in BACHD mice, Ca_v_1.2 mRNA levels are not different when comparing WT and BACHD mice. Then, we suggest that Htt mutation is not affecting Ca_v_1.2 gene expression but protein trafficking or degradation. mHtt could change Ca_v_1.2 cell trafficking and localization, which could prevent Ca_v_1.2 protein degradation. For instance, mHtt may interact with auxiliary subunits of L-type Ca channels, altering channel trafficking. In addition, growing evidence has suggested that expanded polyglutamine repeats facilitate the interactions of mHtt protein with huntingtin-associated proteins selectively expressed in the striatum and cortex and also other proteins that are ubiquitously expressed (Martin et al., 1999). Among these proteins are calmodulin, Huntingtin-associated protein 1 (Hap1), huntingtin protein interacting proteins (Hip1 and 2), and glyceraldehyde-3-phosphate dehydrogenase (Bao et al., 1996, Burke et al., 1996, Li and Li, 2004). Particularly, Hap1 may maintain neuronal transmission and neurotrophic functions by regulating intracellular trafficking, recycling, and stabilization of receptors (Gauthier et al., 2004). Recently, Hap1 was shown to regulate the surface expression level and intracellular trafficking of Ca_v_1.2 channel in INS-1 cells, a pancreatic β-cell line, therefore regulating insulin secretion (Pan et al., 2016). According to these data, impaired Hap1 function is involved in altering the distribution of Ca_v_1.2 on the plasma membrane of INS-1 cells, leading to a decrease in insulin release (Pan et al., 2016). Since Hap1 is highly expressed in the brain, interaction of mHtt protein with Hap1 could induce other protein dysfunctions and consequently lead to the toxicity characteristic of HD (Li et al., 1995). In the future, it would be interesting to investigate whether impaired Hap1 function is involved in the dysregulation of Ca_v_1 or Ca_v_2 channels observed in our model. Thus, to elucidate the underlying mechanism of our findings, further experiments are essential to test the hypothesis that the trafficking of those channels are altered in our model, which could alter Ca_v_1.2 protein degradation.

In conclusion, our results suggest that L-type Ca^2+^ channels are affected in a mouse model of HD. mHtt protein might disrupt calcium homeostasis via upregulation of L-type Ca^2+^ channels, triggering the production of free radicals and oxidative stress, mitochondrial dysfunction, and, eventually, cell death. Finally, *in vitro* concentration–response assay measuring neuronal cell death showed that L-type Ca^2+^ channel blockers were able to reduce neuronal cell death of WT and BACHD cultured neurons (Figure 5). Altogether, the data constitute a previously unrecognized mechanism that may contribute to our understanding of HD pathogenesis. In addition, Ca_v_1 channel blockers should be further investigated as potential therapeutics tools.

## Summary Statement

Our main results show that in a mouse model of Huntington’s disease, Ca_v_1 channels may be dysfunction. Furthermore, L-type Ca^2+^ currents are enhanced in cortical neurons. By using Ca_v_1 antagonists, we were able to observe neuronal protection against glutamate toxicity.
